# Supplementary material for: Core-Shell Carbon Nanofibers@Ni(OH)2/NiO Composites for High-Performance Asymmetric Supercapacitors
Source: Materials (Basel). 2022 Nov 24;15(23):8377. doi: 10.3390/ma15238377 (PMC9738488; doi:10.3390/ma15238377)
Supplement: Supplementary file 1 [file materials-15-08377-s001.zip › materials-2027876-supplementary.pdf]

Supplementary data

Core-Shell Carbon Nanofiber@Ni(OH)$_2$/NiO Composites for High-Performance Asymmetric Supercapacitors

Peizhi Fan and Lan Xu *
National Engineering Laboratory for Modern Silk, College of Textile and Engineering, Soochow University, Suzhou 215123, China
* Correspondence: lanxu@suda.edu.cn

Materials

Polyacrylonitrile (PAN, Mw=150,000) was purchased from Hefei Sipin Technology Co., Ltd. Urea was supplied by Beijing Inoke Technology Co., Ltd. $Ni(CH_3COO)_2{\cdot}4H_2O$ was provided by Utop Technology Suzhou Co., Ltd. $Ni(NO_3)_2{\cdot}6H_2O$ was supplied by Suzhou University. Ultrahigh capacitance activated carbon was provided by Youtepu technology Suzhou Co., Ltd. Polytetrafluoroethylene emulsion (PTFE, 60 wt%) was purchased from Shanghai Rin Technology Development Co., Ltd. N,N-Dimethylformamide (DMF) and ethanol were obtained from Jiangsu Qiangsheng Functional Chemical Co., Ltd. All reagents are of analytical grade and the water used is deionized water.

Characterization

The morphologies of samples were observed by an ultra-high resolution field emission scanning electron microscope (HR-FESEM, S-4700, Regulus 8100) equipped with an energy dispersive X-ray spectrometry (EDS) and a field emission transmission electron microscope (FETEM, TecnaiG2F20). Elemental content and energy spectrum were measured by TM 3030. The crystal structures of samples were analyzed by X-ray diffraction (XRD, D8 Advance, CuKα, scanning speed 2° min^{-1}, 2Theta = 5°-80°) and Raman spectroscopy

(Raman, Xplora Pius, 532 nm laser). The specific surface area and pore size distribution of samples were characterized by a four-station automatic physical adsorption instrument (Micrometrics ASAP460, N_2 adsorption and desorption). The chemical compositions of samples were measured by X-ray photoelectron spectroscopy (XPS, Thermo Scientific K-Alpha, voltage 12 kV, current 6 mA).

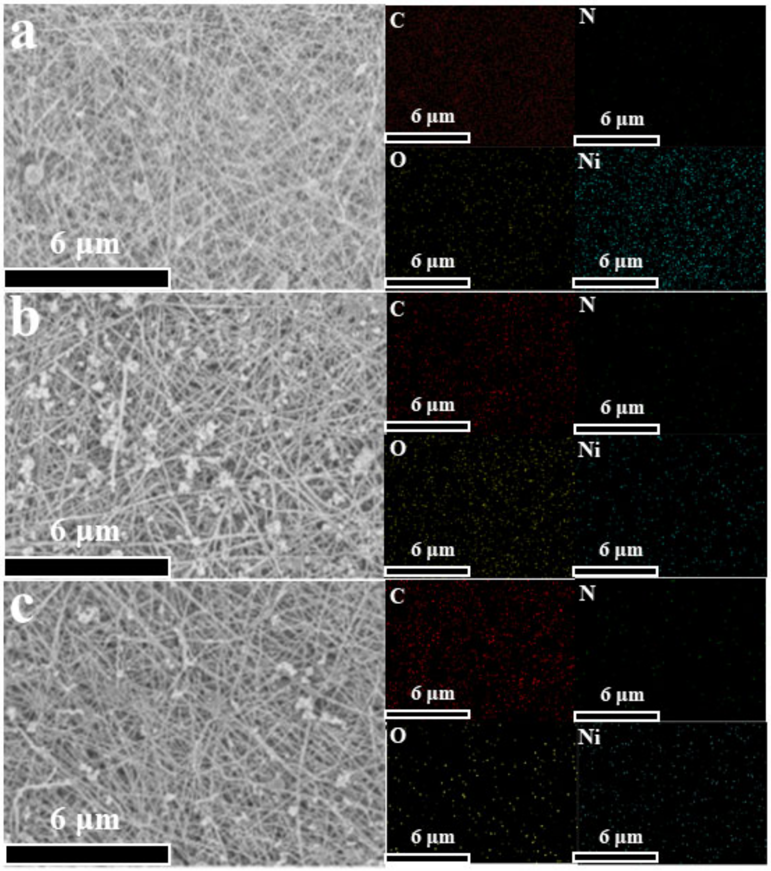

Figure S1. (a)EDS pictures of CNFs@$Ni(OH)_2$/NiO-200. (b) EDS pictures of CNFs@$Ni(OH)_2$/NiO-250. (c) EDS pictures of CNFs@$Ni(OH)_2$/NiO-300.

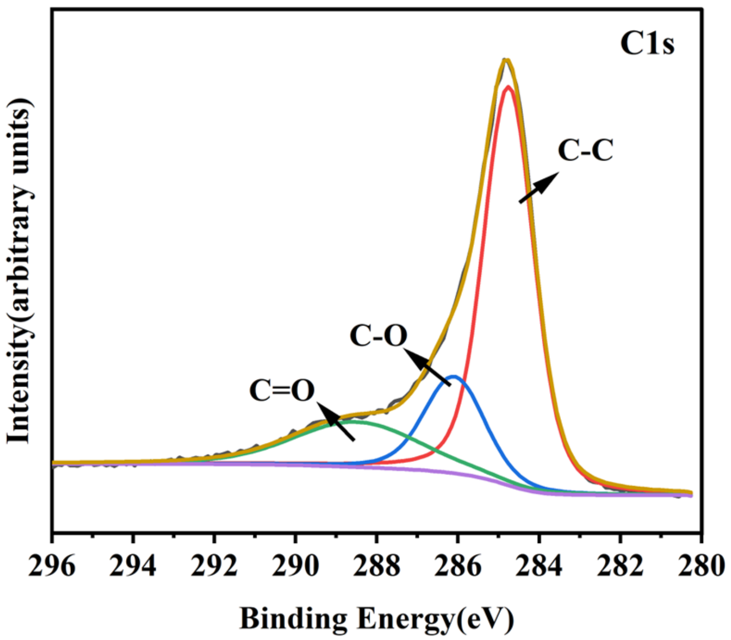

Figure S2. C1s XPS spectrum of CNFs@Ni$(OH)_2$/NiO-250.

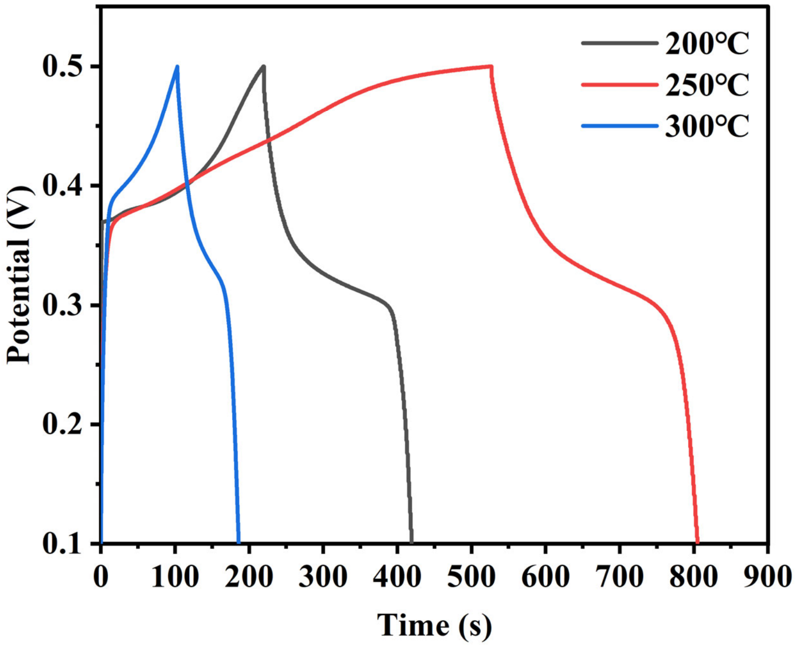

Figure S3. GCD curves of CNFs@Ni$(OH)_2$/NiO-X at 1 A g^{-1} current density.

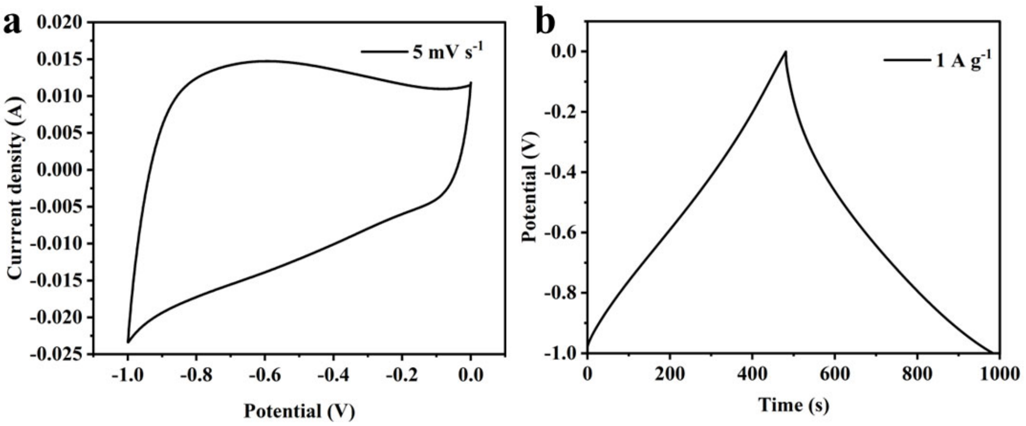

Figure S4. (a) CV curve of AC electrode at 5 mV s^{-1} scan rate; (b) GCD curve of AC electrode at 1 A g^{-1} current density.

Table S1. Comparison of CNFs@$Ni(OH)_2$/NiO-250 with other reported nickel-based electrodes.

Electrode material	Preparation method	Morphology	Specific capacitance	Electrolyte	Refs.
NiO@$Ni(OH)_2$-α-MoO_3	Hydrothermal reaction	Granular	445 F g^{-1} (1A g^{-1})	1 M KOH	[1]
$Ni(OH)_2$/NiO/Ni composite nanotube	Electrodeposition+Electrochemical oxidation	Composite nanotube arrays	1070 F g^{-1} (15A g^{-1})	1 M KOH	[2]
CeO_2@MnO_2 core–shell heterostructures	Hydrothermal reaction	Nanowires	255.2 F g^{-1} (0.25 A g^{-1})	1 M Na_2SO4	[3]
NiO-rGO	Hydrothermal and Hummers method	Nanosheets	343 C g^{-1} (1 A g^{-1})	1 M NaOH	[4]
Co_2SiO_4/$Ni(OH)_2$	Hydrothermal reaction+Water bath heating	Core-shell nanoribbons	1101 F g^{-1} (1 A g^{-1})	3M KOH	[5]
$Ni(OH)_2$/g-C_3N_4/RGO composite	Hydrothermal reaction	3D petal-like	543.8 F g^{-1} (1 A g^{-1})	6M KOH	[6]

$Co(CO_3)_{0.5}(OH)/Ni_2(CO_3)(OH)_2$	Hydrothermal reaction	Nanobelt	987 F g^{-1} (1 A g^{-1})	6M KOH	[7]
Ni-MOF@CNF	Electrospinning+Hydrothermal growth+Carbonization	Petal-like	742.2 F g^{-1} (1 A g^{-1})	3M KOH	[8]
NiO nanoparticles	Annealing Process	Spherical particles	644 F g^{-1} (0.5 A g^{-1})	0.1M KOH	[9]
flowerlike NiO	Homogeneous precipitation method	Petal granules	631.7 F g^{-1} (1 A g^{-1})	6 M KOH	[10]
CNFs@$Ni(OH)_2$/NiO-250	Electrospinning+Hydrothermal process+Carbonization	Fibrous	695 F g^{-1} (1A g^{-1})	3M KOH	This work

References

1. Manibalan, G.; Govindaraj, Y.; Yesuraj, J.; Kuppusami, P.; Murugadoss, G.; Murugavel, R.; Rajesh Kumar, M. Facile synthesis of NiO@Ni(OH)2-α-MoO3 nanocomposite for enhanced solid-state symmetric supercapacitor application. *J Colloid Interf Sci* **2021**, *585*, 505-518, doi:https://doi.org/10.1016/j.jcis.2020.10.032.
2. Dai, X.; Chen, D.; Fan, H.; Zhong, Y.; Chang, L.; Shao, H.; Wang, J.; Zhang, J.; Cao, C.-n. Ni(OH)2/NiO/Ni composite nanotube arrays for high-performance supercapacitors. *Electrochim Acta* **2015**, *154*, 128-135, doi:https://doi.org/10.1016/j.electacta.2014.12.066.
3. Zhu, S.J.; Jia, J.Q.; Wang, T.; Zhao, D.; Yang, J.; Dong, F.; Shang, Z.G.;

Zhang, Y.X. Rational design of octahedron and nanowire CeO2@MnO2 core–shell heterostructures with outstanding rate capability for asymmetric supercapacitors. *Chem. Commun.* **2015**, *51*, 14840-14843, doi:10.1039/C5CC03976B.

4. Gao, X.; Zhang, H.; Guo, E.; Yao, F.; Wang, Z.; Yue, H. Hybrid two-dimensional nickel oxide-reduced graphene oxide nanosheets for supercapacitor electrodes. *Microchem. J.* **2021**, *164*, 105979, doi:https://doi.org/10.1016/j.microc.2021.105979.

5. Zhao, Y.; Zhang, Y.; Cheng, Y.; Zhao, W.; Chen, W.; Meng, C.; Huang, C. Synthesis of Co2SiO4/Ni(OH)2 core–shell structure as the supercapacitor electrode material with enhanced electrochemical properties. *Mater Lett* **2021**, *282*, 128774, doi:https://doi.org/10.1016/j.matlet.2020.128774.

6. Liu, H.; Liu, B.; Sun, X.; Han, X.; Cui, J.; Zhang, Y.; He, W. A simple hydrothermal method for the preparation of 3D petal-like Ni(OH)2/g-C3N4/RGO composite with good supercapacitor performance. *Inorg. Chem. Commun.* **2020**, *122*, 108263, doi:https://doi.org/10.1016/j.inoche.2020.108263.

7. Zhang, G.; Qin, P.; Nasser, R.; Li, S.; Chen, P.; Song, J. Synthesis of Co(CO3)0.5(OH)/Ni2(CO3)(OH)2 nanobelts and their application in flexible all-solid-state asymmetric supercapacitor. *Chem Eng J* **2020**, *387*, 124029, doi:https://doi.org/10.1016/j.cej.2020.124029.

8. Shin, S.; Shin, M.W. Nickel metal–organic framework (Ni-MOF) derived NiO/C@CNF composite for the application of high performance self-standing supercapacitor electrode. *Appl Surf Sci* **2021**, *540*, 148295, doi:https://doi.org/10.1016/j.apsusc.2020.148295.
9. Gunasekaran, S.S.; Gopalakrishnan, A.; Subashchandrabose, R.; Badhulika, S. Phytogenic generation of NiO nanoparticles as green-electrode material for high performance asymmetric supercapacitor applications. *J Energy Storage* **2021**, *37*, 102412, doi:https://doi.org/10.1016/j.est.2021.102412.
10. Shi, H.; Ma, M.; Liu, P.; Jia, X.; Yang, F.; Zhao, B.; Li, Z. Preparation of petal-particle cross-linking flowerlike NiO for supercapacitor application. *Electroanal Chem* **2020**, *876*, 114481, doi:https://doi.org/10.1016/j.jelechem.2020.114481.
